# Ultrasonographic and histopathologic features associated with common ocular diseases in donkeys (*Equus asinus*)

**DOI:** 10.1007/s11259-023-10102-4

**Published:** 2023-03-21

**Authors:** Mona N. Wafy, Elham A. Hassan, Kawkab A. Ahmed, Amal M. Aboelmaaty, Ashraf M. Abu-Seida

**Affiliations:** 1 Department of Surgery, Anesthesiology and Radiology, Faculty of Veterinary Medicine, Cairo University, 12211 Giza, Egypt; 2 Pathology Department, Faculty of Veterinary Medicine, Cairo University, Giza, Egypt; 3 Veterinary Research Institute, National Research Centre, Giza, Egypt

**Keywords:** Eye, Ocular, Donkey, Cataract, Ultrasound, Histopathology

## Abstract

There is a lack of reports describing ultrasonographic and histopathologic features of ocular diseases in donkeys. The present study aimed to document ultrasonographic and histopathologic changes associated with common ocular diseases in donkeys. The study included 45 donkeys (64 eyes) with ocular diseases that had reached the end of their working lives and requested to be used for educational and research purposes. Complete clinical, ophthalmic, ultrasonographic, gross pathologic and histopathologic examinations were included. Ocular abnormalities were documented, tabulated, and analyzed. Seventy-five ocular abnormalities were diagnosed with multiple involvements within the same eye including anterior uveitis (*n* = 13; 22.8%), cataract (*n* = 57; 76%), retinal detachment (*n* = 3; 4%), lens and phthisis bulbi (*n* = 2; 2.6%). Descriptive ultrasonographic findings of ocular abnormalities were included. Gross pathologic and histopathology findings confirmed the ultrasonography findings. Ultrasonography provided a clinically useful tool offering insight into detailed intraocular structures especially with opacification of the dioptric structures of the eye.

## Introduction

The domesticated donkey plays an important role in the social and economic activities of human life. Despite the extensive development in technology and mechanical engineering, donkeys are still used for agricultural and farming activities, transportation, and power activities in most rural areas. It is a main source of meat, milk and hide production (Seyiti and Kelimu 2021).

Ocular diseases have been previously reported in donkeys including corneal lacerations, keratitis, lens luxation, uveitis, and neoplasms (Misk 1990; Bradley et al. 2020). Ocular examination with visualization of the intraocular structures is very crucial for accurate diagnosis, prognosis and for treatment planning of ocular diseases (Mettenleiter 1995; Penninck et al. 2001; Ramirez and Tucker 2004). Ocular evaluation is challenging under field practice in rural areas especially with presence of opacity within the cornea, aqueous humor, lens, or vitreous humor, or with presence of eyelid swelling obscuring proper examination (Mettenleiter 1995).

Ocular ultrasonography (OU) is a readily available, cost-effective, safe, and noninvasive diagnostic tool that can be performed in equine patients without sedation for complete ophthalmologic examination (Mettenleiter 1995; Dietrich 2007; Wafy et al. 2021). It allows visualization of structures that cannot be observed by use of routine ophthalmic examinations such as retrobulbar structures (Scotty 2005; Gallhoefer et al. 2013; Blohm et al. 2020), can differentiate solid and cystic periocular lesions and helps in target diagnostic sampling through fine needle biopsy sampling (Scotty 2005; Hallowell and Bowen 2007; Valentini et al. 2010).

Several studies have documented the beneficial role of OU in diagnosis of eye disorders in equine such as; keratitis/keratouveitis, corneal edema, corneal ulceration/perforation, stromal abscess, hypopyon, hyphema, recurrent uveitis, glaucoma, intraocular cysts or masses, iris melanomas, irideal cysts, micro-phakia, cataract, lens sub/luxation, viteral opacities, optical nerve neuritis, retinal detachment, endophthalmitis, buphthalmos, microphthalmia, retrobulbar mass and others (Miller and Cartee 1985; Freestone et al. 1989; Whitcomb 2002; Scotty 2005; Hallowell and Bowen 2007; Valentini et al. 2010; Gialletti et al. 2018; Slenter et al. 2020; Abu-Seida et al. 2021).

Although horses and donkeys belong to the family equidae, anatomical differences between them are well documented (Herman 2009). For instance, previous studies revealed that donkey’s eye is substantially smaller than the horse’s in terms of ocular dimensions which should be carefully considered to avoid misinterpretation of donkey’s eye (Laus et al. 2014; Wafy et al. 2021). Limited studies are available documenting the normal ultrasonographic features of the donkey’s eye (Laus et al. 2013, 2014; Salavati et al. 2017; Wafy et al. 2021). None of these studies reported the ultrasonographic features of ocular diseases in donkeys nor the histopathological changes associated with the ongoing diseases. The aim of the present study was to report the correlation between ultrasonographic and histopathologic changes associated with common ocular diseases in donkeys.

## Materials and methods

### Animals

The study was conducted on donkeys diagnosed with ocular diseases that were admitted to the Department of Surgery, Anesthesiology and Radiology - Faculty of Veterinary Medicine, Cairo University, Egypt during the academic years (2018–2021) to be used for educational purposes after termination of their working life as draft animals used for farming and transportation. The donkeys were discarded from work due to variety of causes (senility, loss of vision, limb deformities including incurable diseases affecting bones and joints). Complete clinical, ophthalmic and ultrasonographic examinations were performed by Faculty Staff members (ophthalmologists and radiologists) at the Department of Surgery, Anesthesiology and Radiology (MNW, AMA, EAH and AMA). Gross pathologic and histopathological examinations were also performed by a Faculty Staff member at the Department of Pathology (KAA) just following humane euthanasia of these donkeys to be used as cadavers for practical sessions.

The Institutional Animal Care and Use Committee at Faculty of Veterinary Medicine, Cairo University, Egypt approved this study (Vet.CU.IACUC#VetCU24112020250). All legal and ethical international measures have been fulfilled regarding humane management of the donkeys included in the study.

### Clinical examination

Complete ophthalmic examinations were performed on both eyes without sedation or nerve blocks, initially in ambient barn light followed by assessment in a dark room (Wafy et al. 2021; Ali et al. 2022). Neuro-ophthalmic examination and subjective assessment of vision (menace response, dazzle reflex, maze testing, pupillary light reflex, palpebral reflex), slit lamp examination (Heine® Slit Lamp HSL 150, Mumbai, India), distant and close direct ophthalmoscopy (Riester® Ri-Scope L ophthalmoscope, Vision Tech, Vadodara, India), indirect ophthalmoscopy (Heine Omega 150 with Volk Pan-Retinal® 2.2 hand lens, Volk Optical Inc., Mumbai, India) were performed for all donkeys. Schirmer Tear Testing (I-Schirme® Schirmer Tear Test, Eye Care Products, New Delhi, India) and measurement of intraocular pressure (applanation tonometry, Tonopen XL®, Reichert Technologies, NY, USA) were performed only when deemed necessary.

### Ocular ultrasonography

A standardized trans-palpebral B-mode ultrasound scanning was performed using10-12-MHz linear transducers (SonoVet R3, Samsung Medison, South Korea). Examination was performed while donkeys were manually restrained while keeping the head in an upright position.

A sterile coupling gel (Aquasonic®, Parker Laboratories Inc., New Jersy, USA) was gently rubbed over the closed eyelids to eliminate air bubbles stuck between the eyelid and the transducer. Transverse and sagittal scans were performed as previously described (Laus et al. 2013, 2014; Salavati et al. 2017; Wafy et al. 2021). Methodical examination of the cornea, anterior chamber, iris and ciliary body, lens, vitreous humor, ocular fundus and retrobulbar space was carried out. All abnormalities within the globe’s internal structures were recorded.

At the end of examination, the coupling gel was cleaned from the eye with a piece of cotton and the eyes were carefully washed with clean saline solution, followed by the application of artificial tears.

### Gross pathologic and histopathological examination

Just following humane euthanasia of the donkeys, both eyes were enucleated, photographed, and collected for histopathological examination. Eye samples were fixed in neutral buffered formalin 10%, routinely processed and embedded in paraffin wax. Paraffin blocks were sectioned at 4 μm thickness, deparaffinized with xylene, rehydrated through graded alcohols and stained with Haematoxylin and Eosin (H&E) for subsequent histopathological examination using light microscope (Olympus BX50, Tokyo, Japan).

## Results

The study included 45 donkeys of both sex (26 male and 19 female). These donkeys were skeletally mature animals (median age 9.8, range 4–20 years old) with mean body condition score 4.1 ± 1.2 based on 9-point scale at the end of their working life.

Clinical and ultrasonographic examination revealed that 26 donkeys (57.8%) had unilateral ocular involvement, while the remaining 19 donkeys (42.2%) had bilateral ocular involvement. A total of 75 ocular abnormalities were diagnosed in 64 diseased eyes with multiple involvements within the same eye.

The reported ocular abnormalities included anterior uveitis (13/75, 17.3%), cataract (57/75, 76%), retinal detachment (3/75, 4%), and phthisis bulbi (2/75, 2.6%).

### Anterior uveitis

Anterior uveitis was diagnosed in 13 eyes that were also diagnosed with cataract.

#### Clinical examination

Anterior uveitis was characterized clinically by the presence of ocular pain (blepharospasm) and discharge (epiphora), as well as findings that are consistent with anterior uveitis such as chemosis (10/13 eyes, 76.9%), mitotic pupil (11/13 eyes, 84.6%), corpora nigra atrophy (12/13 eyes, 92.3%), aqueous flare (11/13 eyes, 84.6%), and keratic precipitates (11/13 eyes, 84.6%). Other less common findings associated with anterior uveitis was vitreal opacity in association with cataract (9/13, 69.2%). Vitreal opacity was described as small mobile flakes that appeared floating within the vitreous. Hypopyon was found in two eyes (2/13, 15.4%) with impairment of vision where opacification in the anterior chamber behind the cornea was visualized with whitish material and fibrin threads.

#### Ultrasonographic examination

**Fig. 1:**
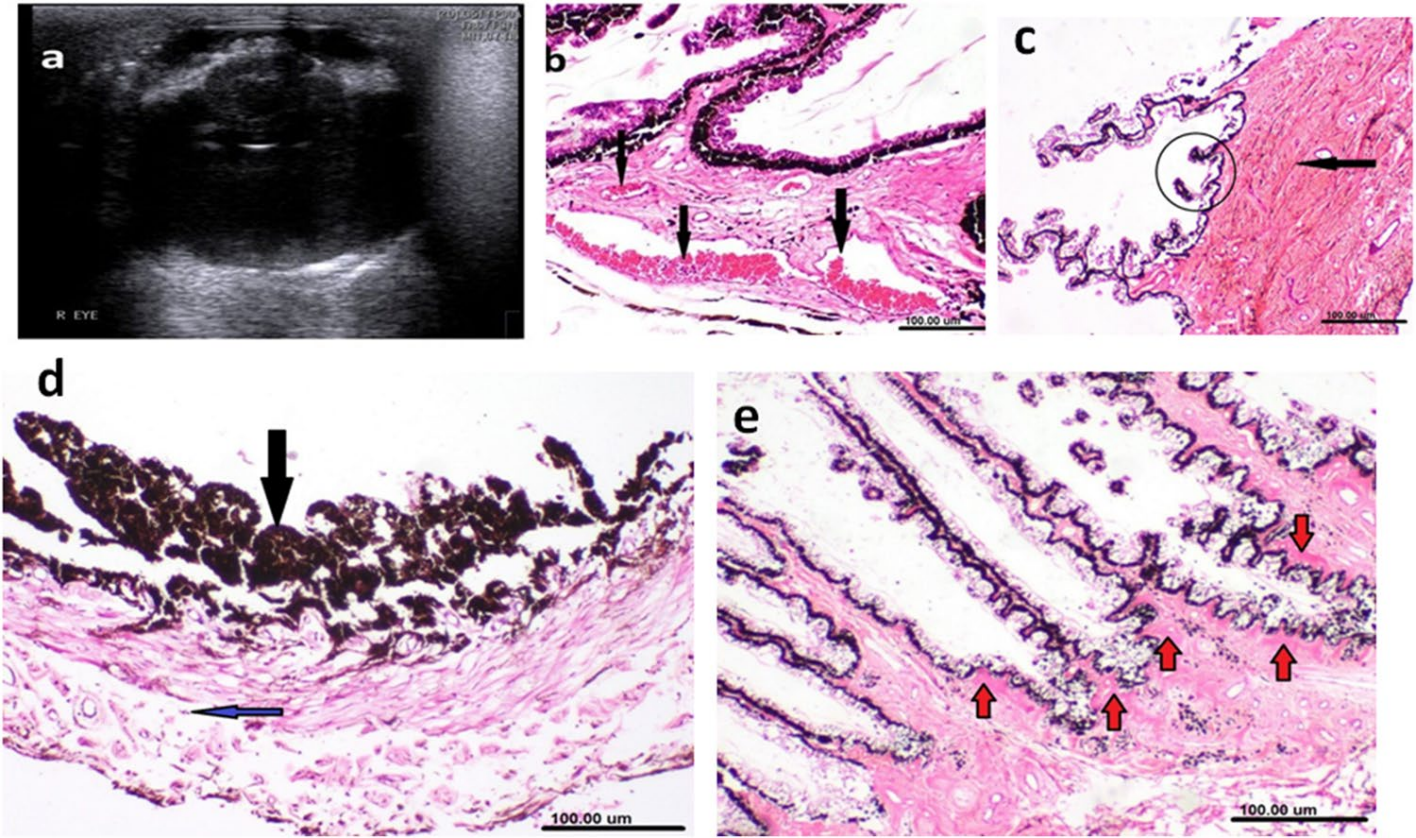
Ultrasonographic (**a**) and histopathologic (**b**-**e**) changes associated with anterior uveitis. Ultrasonography showing hyperechoic iris and ciliary body associated with mature cataract (**a**). Histopathologic examination of ciliary body and processes revealed marked dilatation and congestion of blood vessels [black arrows] (**b**), fibrosis of the ciliary body [black arrow] and short atrophied ciliary processes [circle] (**c**), and hyperplasia of pigmented cell epithelium (black arrow) with few mononuclear inflammatory cells infiltrating the stroma [blue arrow] (**d**), Deposition of amyloid-like eosinophilic material (Cooley bodies) was seen along the non-pigmented ciliary epithelium (**e**). (H&E; scale bar 100 μm)

**Fig. 2:**
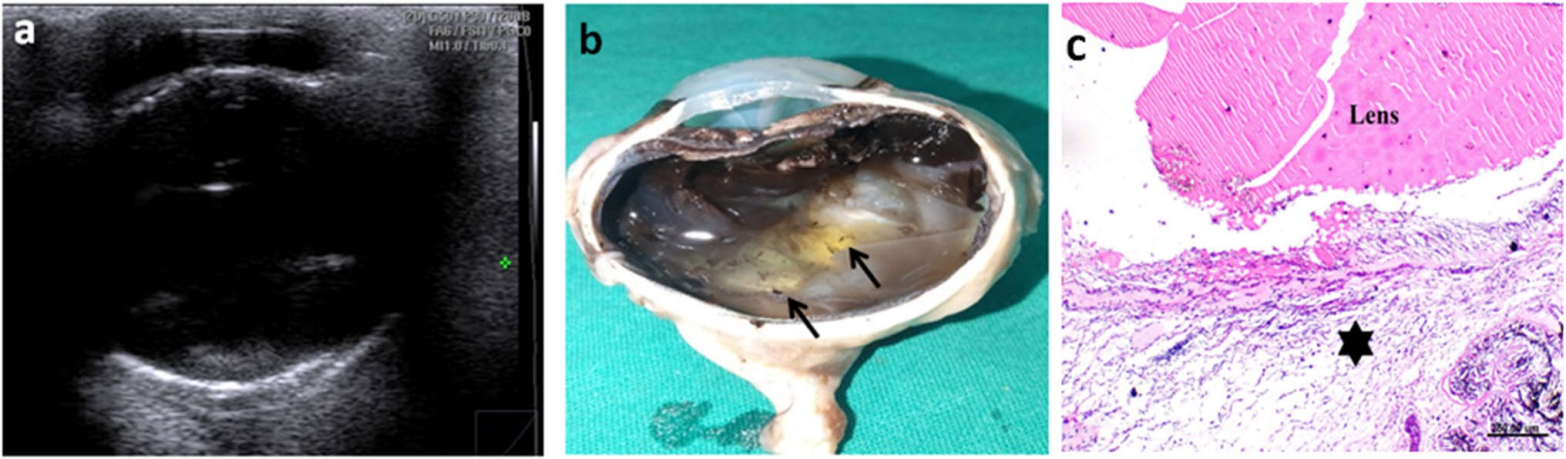
Ultrasonographic (**a**), gross pathologic (**b**) and histopathologic **(c)** changes associated with viterous opacity. **a** Ultrasonography showing echogenic materials within the anechoic vitreous and hyperechoic mature cataractous lens. **b** Cross sectional examination of the eye showing minute black flakes (arrows) floating within the vitreous. **c** The vitreous is composed of widely separated spindle cells and infiltrated with mononuclear cells (asterisk)

Iridocyclitis was visualized as increased hyperechogenicity of iris and ciliary body (Fig. 1a). Vitreous opacities could be distinguished by the presence of hyperechoic structures within the anechoic vitreous (Fig. 2a). The presence of such echogenicities suggests being hemorrhage, white blood cells, or fibrin which was confirmed during sectioning of the eye and gross pathologic examination (Fig. 2b).

One case had hypopyon and lens capsule rupture with cataract formation. The hypopyon was visualized as hyperechogenic material in the anechoic anterior chamber. Cataract in the lens was visualized as an encircled echogenic structure which appeared herniated from the ruptured lens capsule.

#### Histopathologic examination

The ciliary body had marked dilated, congested blood vessels and a thick membrane of hyaline, eosinophilic material lining the ciliary epithelium. Edema and fibrosis of the ciliary body and short atrophied ciliary processes were also seen. The iris had hyperplasia of pigmented cell epithelium, slight edema and few mononuclear (macrophages, lymphocytes, and plasma cells) inflammatory cells infiltrating the stroma (Fig. 1b-d). Deposition of amyloid-like eosinophilic material (Cooley bodies) was seen along the non-pigmented ciliary epithelium (Fig. 1e). The vitreous was composed of widely separated spindle cells and infiltrated with mononuclear cells (Fig. 2c). Histopathologic examination of hypopyon was characterized by accumulation of purulent exudate in the anterior chamber (suppurative uveitis) associated with massive inflammatory cell infiltration (mainly neutrophils, macrophages and lymphocytes) and fibroblasts proliferation. Associated cataract with liquefaction of the lens and neutrophils infiltration was noticed. Detachment of descemet membrane and focal spindle cell proliferation were seen on the corneal stromal with fibrosis and inflammatory cell infiltration. The choroid had massive inflammatory cell infiltration (neutrophils, macrophages, lymphocytes and few eosinophils), purulent exudate, fibrosis and thickened, hyalinized blood vessel’s wall. The iris exhibited marked edema and massive inflammatory cell infiltration.

### Cataract

#### Clinical examination

Clinical examination of donkeys with cataract (57 eyes) revealed presence of varying degrees of lens opacities (13/57, 22.8% incipient cataract, 16/57, 28.1% immature cataract, 26/57, 45.6% mature cataract, and 2/57, 3.5% hypermature cataract). No defect in functional vision could be detected in 29 eyes while in the remaining 28 eyes (mature and hypermature cataract) the vision appeared to be affected during examination.

#### Ultrasonographic examination

Ultrasonographically, the lens was changed in shape with presence of anterior and posterior crystalloids. Lens echogenicity was markedly changed and transformed from anechoic to echodense structure. The degree of echogenicity varied greatly depending upon the chronicity, maturity and extent of the lens affected (cortical, nuclear, or total lens cataract). Different degrees of cataract were identified by ultrasonography especially in mature and hypermature cataract.

**Fig. 3:**
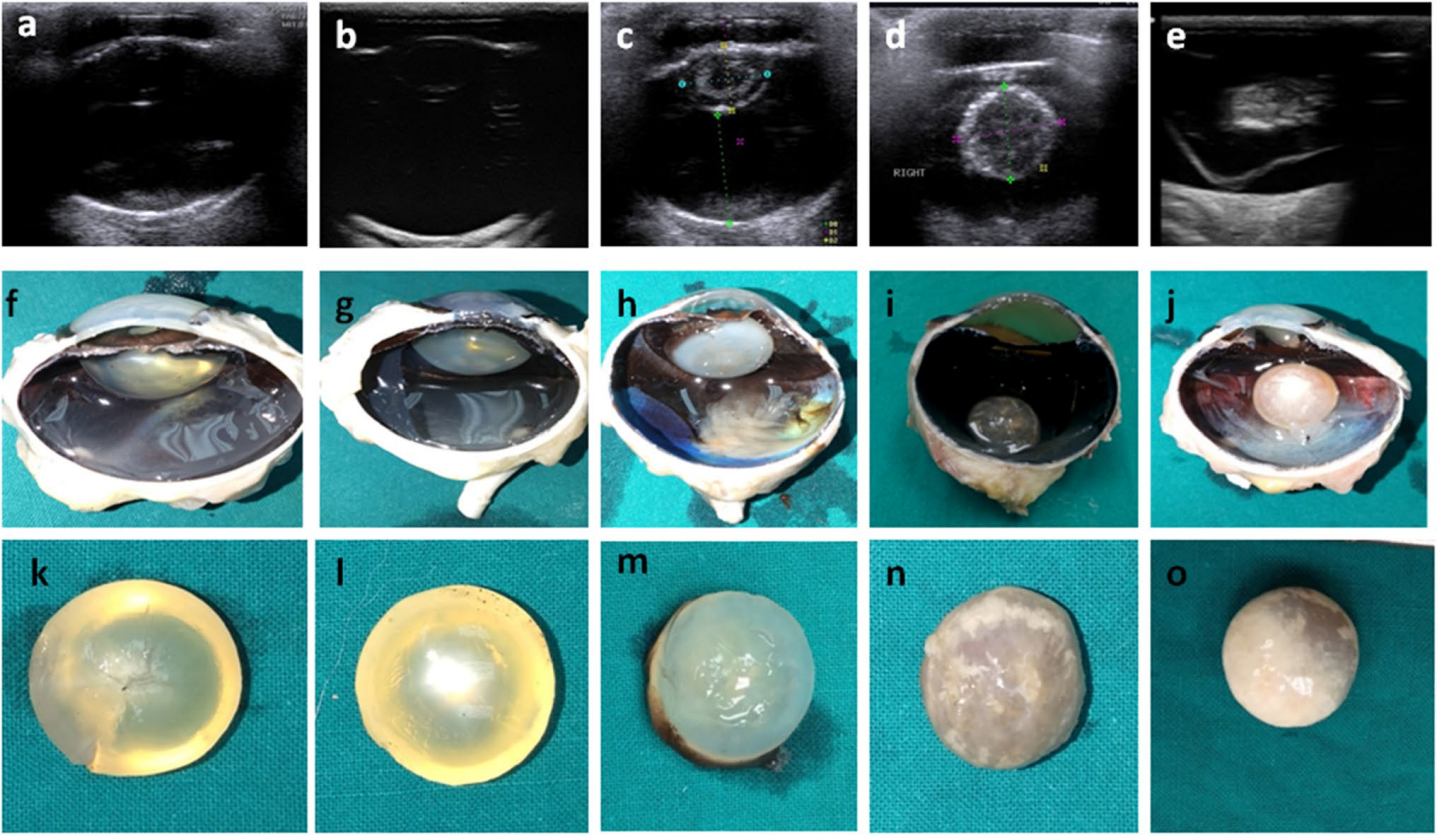
Ultrasonographic (upper row), gross pathologic (middle row), and lenticular changes (lower row) associated with different forms of cataract. Ultrasonograms of incipient cataract showing small hyperechoic areas of lens nucleus (**a**), immature cataract showing increased hyperechoic areas of lens nucleus > 5% of lens nucleus (**b**), mature cataract showing two hyperechoic circles within the lens indicating nuclear and capsular cataract (**c**), hypermature cataract showing completely hyperechoic lens with increase in the thickness of lens [hypermature intumescent cataract] (**d**) or decrease in the size of lens [hypermature resorbed cataract] (**e**). Gross pathologic (**f**-**J**) and lenticular (**k**-**o**) findings are compatible the ultrasonographic findings where lens opacity increases with advancement of cataract. The lens becomes more spherical and with irregular capsule at later stages of cataract

**Fig. 4:**
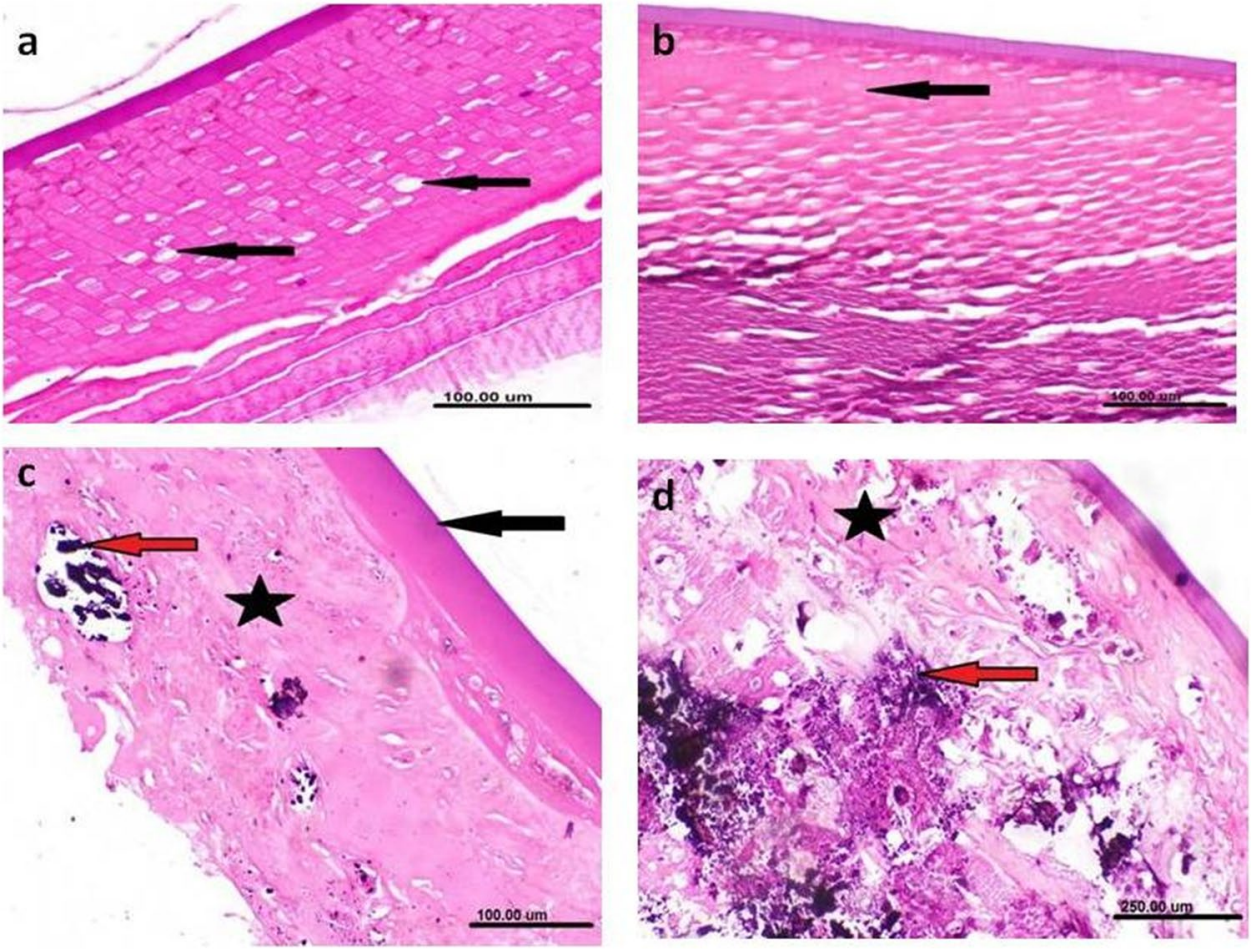
Histopathological changes associated with different stages of cataract. **a** Swelling and vacuolation of lens fibers with absence of their nuclei (black arrows). **b** Homogenous eosinophilic lenticular mass, vascularization of some lens fibers and disappearance of their nuclei (black arrow). **c** Increased thickness of lens capsule (black arrow), homogenous eosinophilic liquefied cortex (asterisk) and mineralization of deep cortical layer (red arrow). **d** Increased thickness of lens capsule, homogenous eosinophilic liquefied fragmented lenticular mass (asterisk) and marked mineralization of the deep cortical layer (red arrow). (H&E; scale bar 100 μm)

Ultrasonographic, postmortem and lenticular changes as well as histopathological changes associated with different grades of cataract are demonstrated in Figs. 3 and 4, respectively.

Incipient cataract represented 13/57 (22.8%) of eyes with cataractous lens where hypoechogenicity was only detected in a small portion of lens nucleus without any remarkable changes in lens capsule echogenicity or change in lens size. Histopathological sections of eyes with incipient cataract revealed swelling and vacuolation of lens fibers.

Immature cataract was diagnosed ultrasonographically in 16/57 (28.1%) of cataractous lenses where the echogenic area occupying the lens was seen in more than 5% of the lens nucleus. Immature cataract was mainly nuclear cataract where echogenicity increased with maturity. Histopathological sections of immature cataract demonstrated swelling and vacuolization of the lens fibers with absence of their nuclei.

Mature cataract was diagnosed in most of the donkeys with cataract (*n* = 26/57; 45.6%). Ultrasonographically, the lens content could be distinguished by presence of two hyperechoic circular structures in cortical cataract and nuclear mature cataract. The outer circle represented the area of the lens cortex while the inner circle representing the peripheral part around the nucleus. In total mature cataract the entire lens material appeared completely echogenic. Histopathological examination revealed homogenous eosinophilic lenticular mass and vacuolization of some lens fibers with absence of their nuclei.

Hypermature cataract was recorded in 2/57 (3.5%) of the cataractous lenses. The lens appeared ultrasonographically as a completely hyperechoic structure. Both lens capsules were thickened and could be seen distinctly in the same scan. The lens itself had expanded in size and became more spherical in one eye with intumescent cataract, while the lens shrank in size, and the lens capsules had an unusual irregular shape in another animal with resorbed cataract. Histopathological sections of hypermature intumescent cataract revealed thickening of lens capsule, homogenous eosinophilic liquified cortex and mineralization of the deep cortical layer. The choroid exhibited marked dilatation and congestion of blood vessels.

Resorbed cataract had the same pathologic findings present in hypermature intumescent cataract, short atrophied ciliary processes with vacuolated epithelium, fragmented lenticular mass and marked mineralization of the deep cortical layer.

**Fig. 5:**
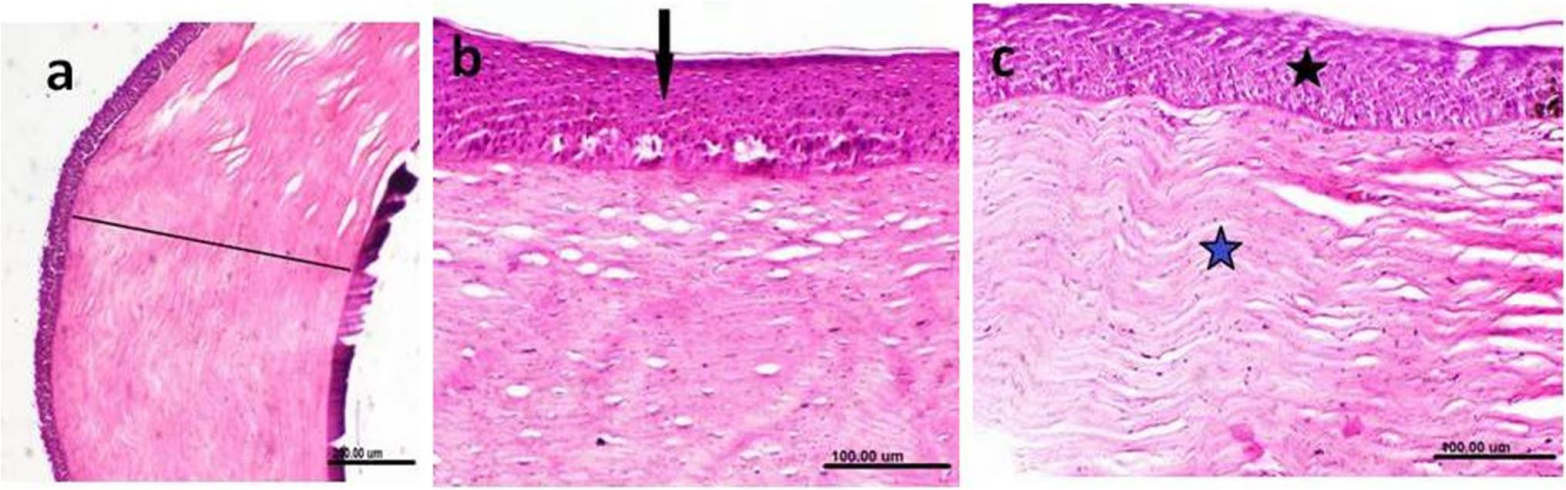
Photomicrographs of the cornea obtained from cataractous eye. The cornea demonstrated marked thickening of corneal epithelium (**a**), thickening and hypertrophy of corneal epithelium [black arrow] (**b**) and thickening and hypertrophy of corneal epithelium (black asterisk) as well as thickening of the corneal stroma [blue asterisk] (**c**)

Most eyes diagnosed clinically and ultrasonographically with cataract had associated histopathological changes involving the iris, ciliary body and process, retina, choroid and sclera (Fig. 5).

**Fig. 6:**
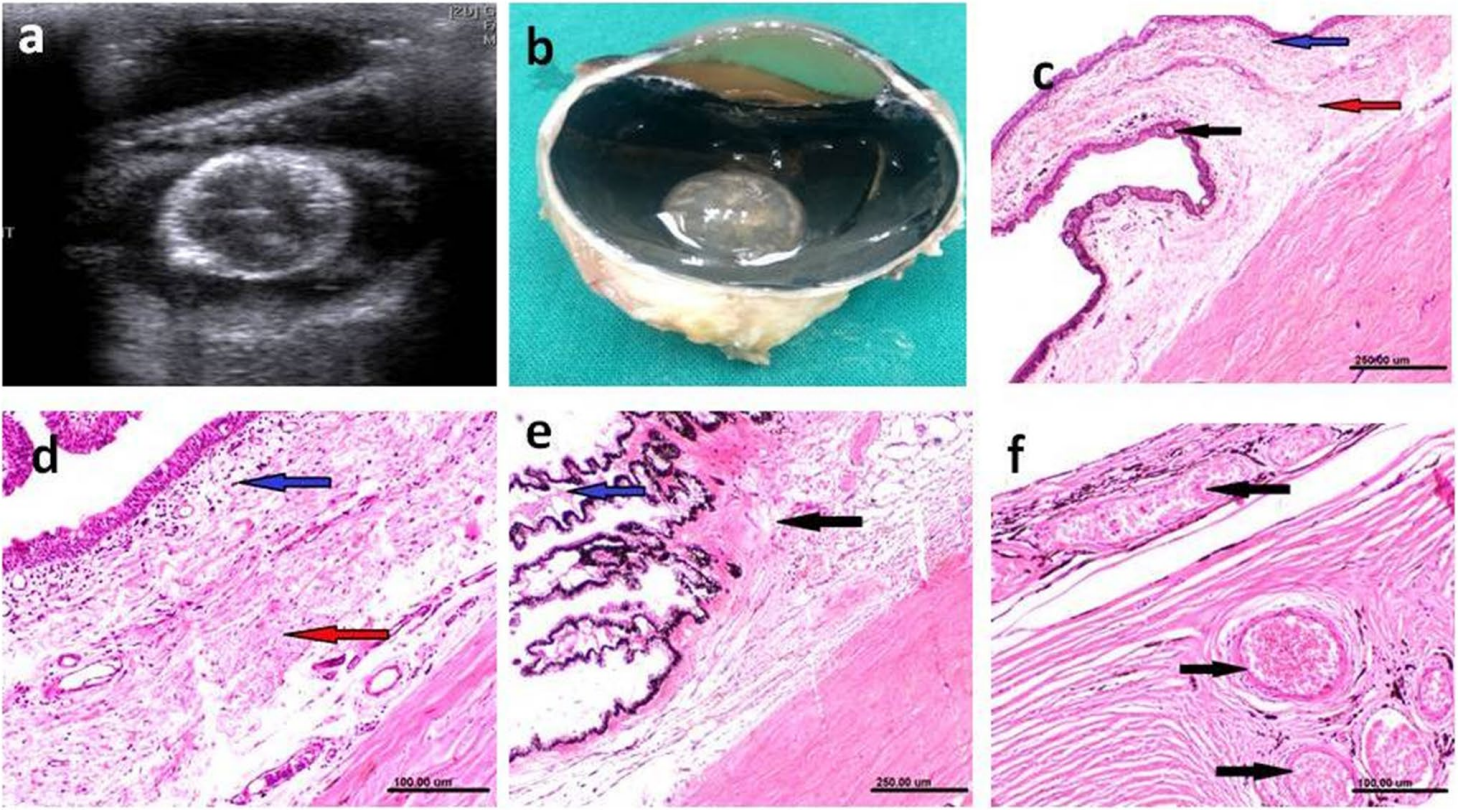
Ultrasonographic (**a**), gross pathologic (**b**) and histopathologic (**c**-**f**) changes of an eye with lens luxation associated with hypermature cataract and iridocyclitis. **a** The lens showing echogenic hypermature cataract and posterior luxation. **b** Cross section of the same eye showing the lens floating into the vitreous. **c**,**d** Histopathological sections of the sclera showing vacuolar degeneration of the epithelial lining [black arrow], edema [red arrow] and inflammatory cell infiltration [blue arrow]. **e** Marked edema was seen along the ciliary body [black arrow] and process [blue arrow]. **f** The choroid showing marked dilatation and congestion of blood vessels [black arrows]. (H&E scale bar 100 μm)

Lens luxation was diagnosed clinically in two eyes with complete loss of vision. Ultrasonographic examination of an eye with luxated lens revealed that the lens was dislocated from its zonular attachments and located in the posterior segment. The iris leaflets were prominent anterior to the luxated lens (Fig. 6a). Post mortem findings of the enucleated eye confirmed the ultrasonography findings (Fig. 6b). histopathologic examination revealed thickening of lens capsule, homogenous eosinophilic liquified cortex and mineralization of the deep cortical layer. Fibrosis of the ciliary body and short atrophied ciliary processes were also reported. Edema of the ciliary body and ciliary processes marked thickening of the corneal stroma, vacuolar degeneration of sclera epithelial lining and inflammatory cell infiltration were also seen (Fig. 6c-f).

**Fig. 7:**
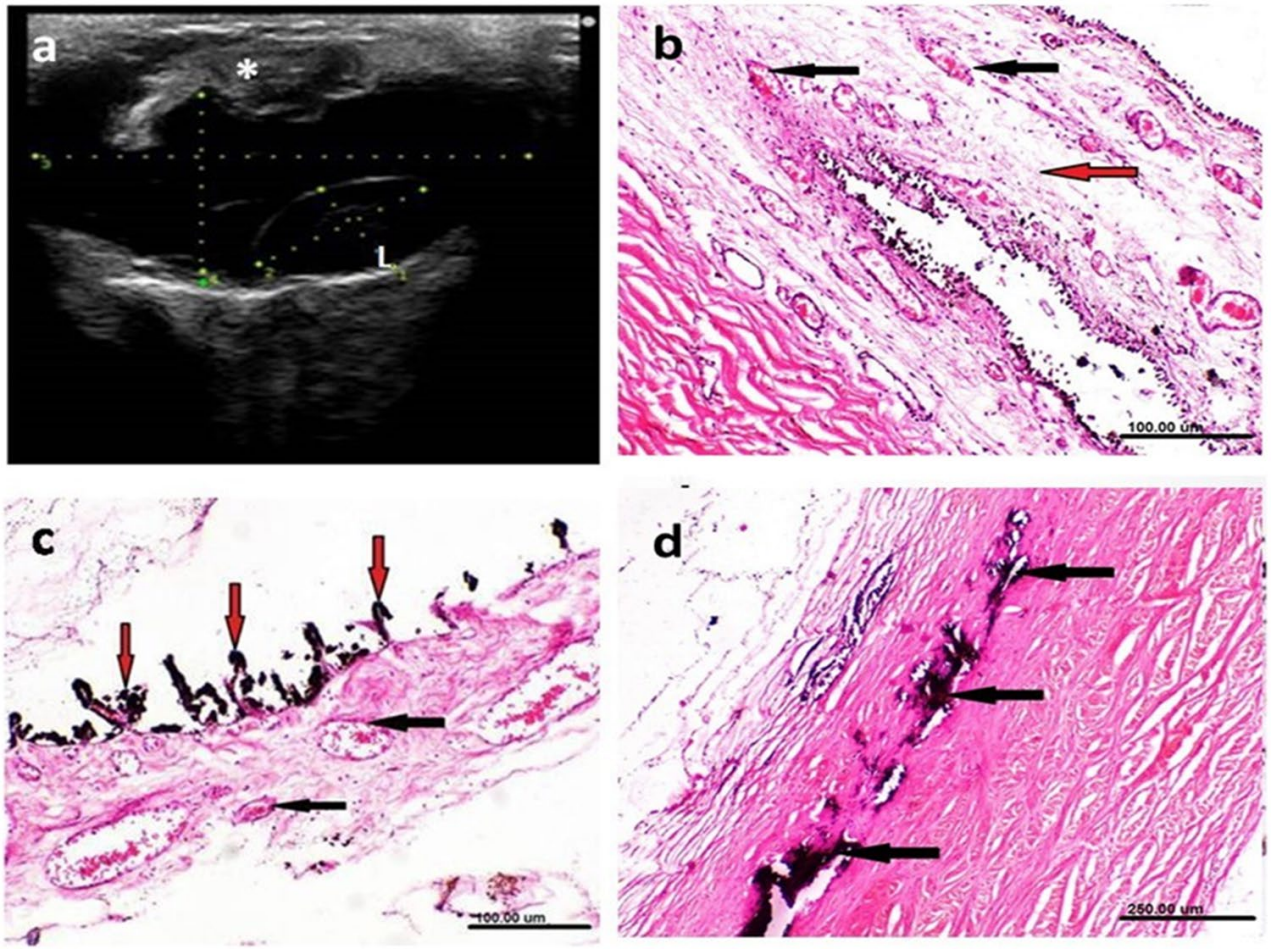
Ultrasonographic (**a**) and histopathologic (**b**-**d**) changes associated with lens luxation caused by a needle penetration. **a** Ultrasonography showing hyperechoic iris and ciliary body (astrisk) and luxated lens (L) in the vitereous chamber. Histopathology showing: **b** congestion of the blood vessels of the iris [black arrows] with marked edema [red arrow] (X100, H&E, scale bar, 100 μm), **c** dilatation and congestion of blood vesseles of the ciliary body [black arrows] with atrophy of the ciliary process [red arrows] (H&E, scale bar, 100 μm), and **d** focal calcification of the sclera [black arrows]. (H&E, X 40, scale bar, 250 μm)

The second eye with lens luxation was seen in association with a needle penetrating the eye as indicated by history. Ultrasonography was carefully made with caution where the iris and ciliary body were ruptured and the crystalline lens was dislocated in the vitreous chamber in association with a needle penetrating the eye (Fig. 7a). Histopathologic examination revealed marked edema and congestion of blood vessels of the iris. Dilatation and congestion of the ciliary body and processes with short atrophied ciliary processes and focal calcification of the sclera (Fig. 7b-d).

### Retinal detachment

**Fig. 8:**
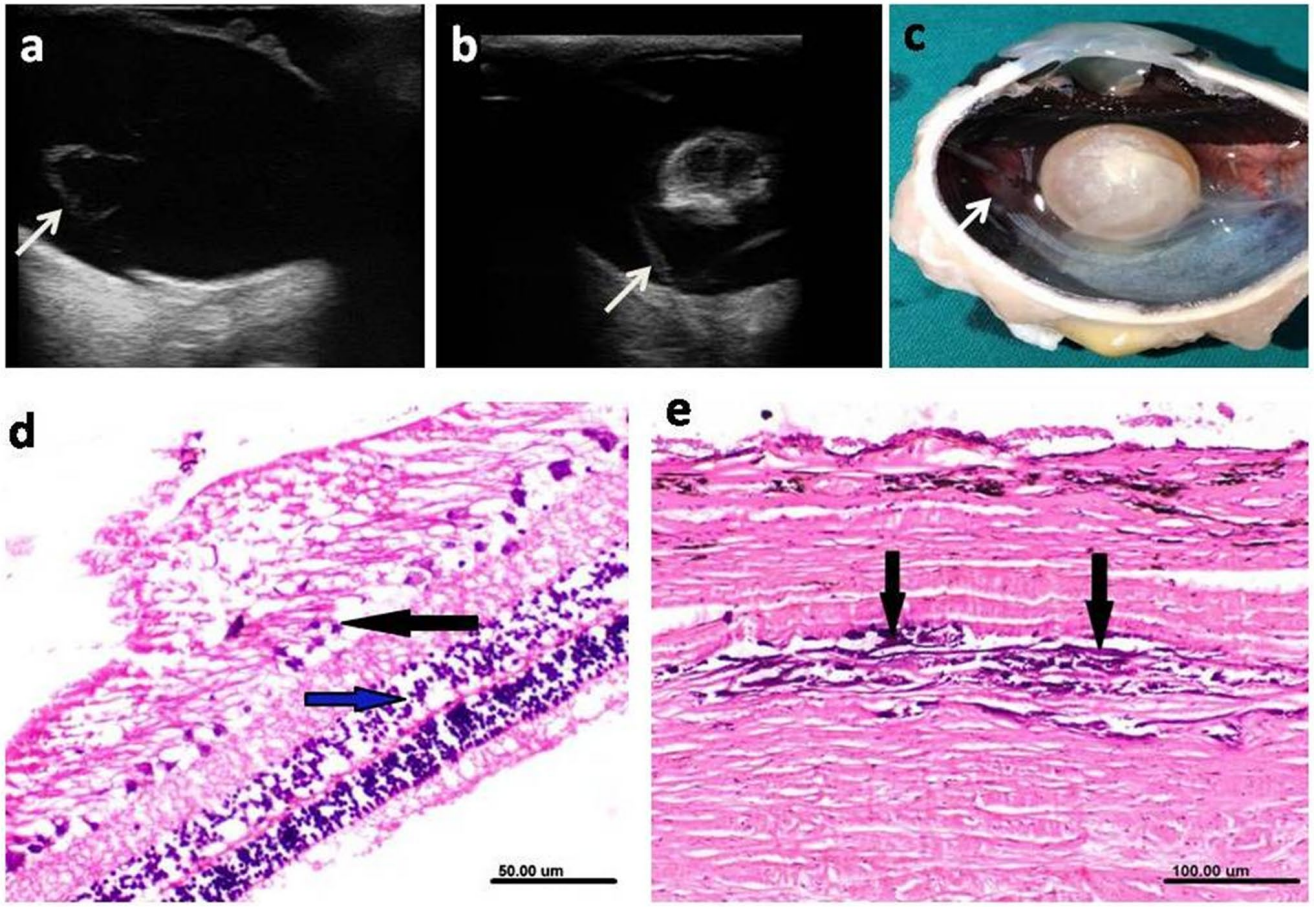
Ultrasonographic (**a**,**b**), gross pathologic (**c**) and histopathologic (**d**,**e**) changes associated with retinal detatchment. **a** Partial retinal detachment showing hyperechoic elevation of the posterior segment (white arrow) with presence of anechoic fluid under this echogenic line. **b** Complete retinal detachment where the retina is only attached to the optic disc and at the ora ciliaris retinae giving a characteristic seagull wings. **c** Grossly, the retina appears separated from its underneath attachment with accumulation of subretinal fluid (white arrow). **d** The retina showing degeneration of both ganglionic cell (black arrow) and nuclear cell (blue arrow) layers (H&E; scale bar 50 μm). **e** The underneath choroid and sclera showing focal calcification of the sclera (black arrows) with hypertrophy of the retinal pigment epithelium. (H&E; scale bar 100 μm)

Partial and complete retinal detachment was diagnosed in two (2.7%) donkeys and one (1.3%) donkey respectively (Fig. 8a, b).

#### Clinical examination

Partial retinal detachment could be diagnosed during ophthalmoscopic examination where part of the retina was removed from its attachment on the posterior segment. The retina appeared partially separated from its underneath attachment with presence of subretinal fluid (Fig. 8c). Complete retinal detachment was associated clinically with complete loss of vision.

#### Ultrasonographic examination

A hyperechoic structure with presence of anechoic fluid under echogenic line was seen with retinal detachment (Fig. 8a). Complete retinal detachment was characterized ultrasonographically by the presence of seagull wings where the retina was only attached at the level of optic disc posteriorly and at the ora ciliaris retinae anteriorly with light grade of mobility (Fig. 8b).

#### Histopathologic examination

Partial retinal detachment appeared as retinal degeneration demonstrated by degeneration of ganglionic cells and both inner and outer nuclear cell layers. Examined sections showed thickening, hypertrophy and vacuolar degeneration of corneal epithelium, homogenous eosinophilic degenerated lenticular mass, focal calcification of the sclera and hyperplasia of ciliary processes (Fig. 8d). In eye with complete retinal detachment, the retina was truely detached and not appeared during the processing of the specimen. The presence of subretinal exudate and hypertrophy of the retinal pigment epithelium were prominent finding. Histopathological changes were seen within the eye with detached retina such as cataract in which the lens exhibited thick capsule, homogenous eosinophilic liquefied lenticular mass, and mineralization of the deep cortical layer. Additionally, the ciliary processes were short and atrophied with vacuolated epithelium.

### Phthisis bulbi

Phthisis bulbi was diagnosed in two eyes (2.7%) where the eye lost its normal appearance, appeared nonfunctional with enophthalmos.

#### Clinical examination

**Fig. 9:**
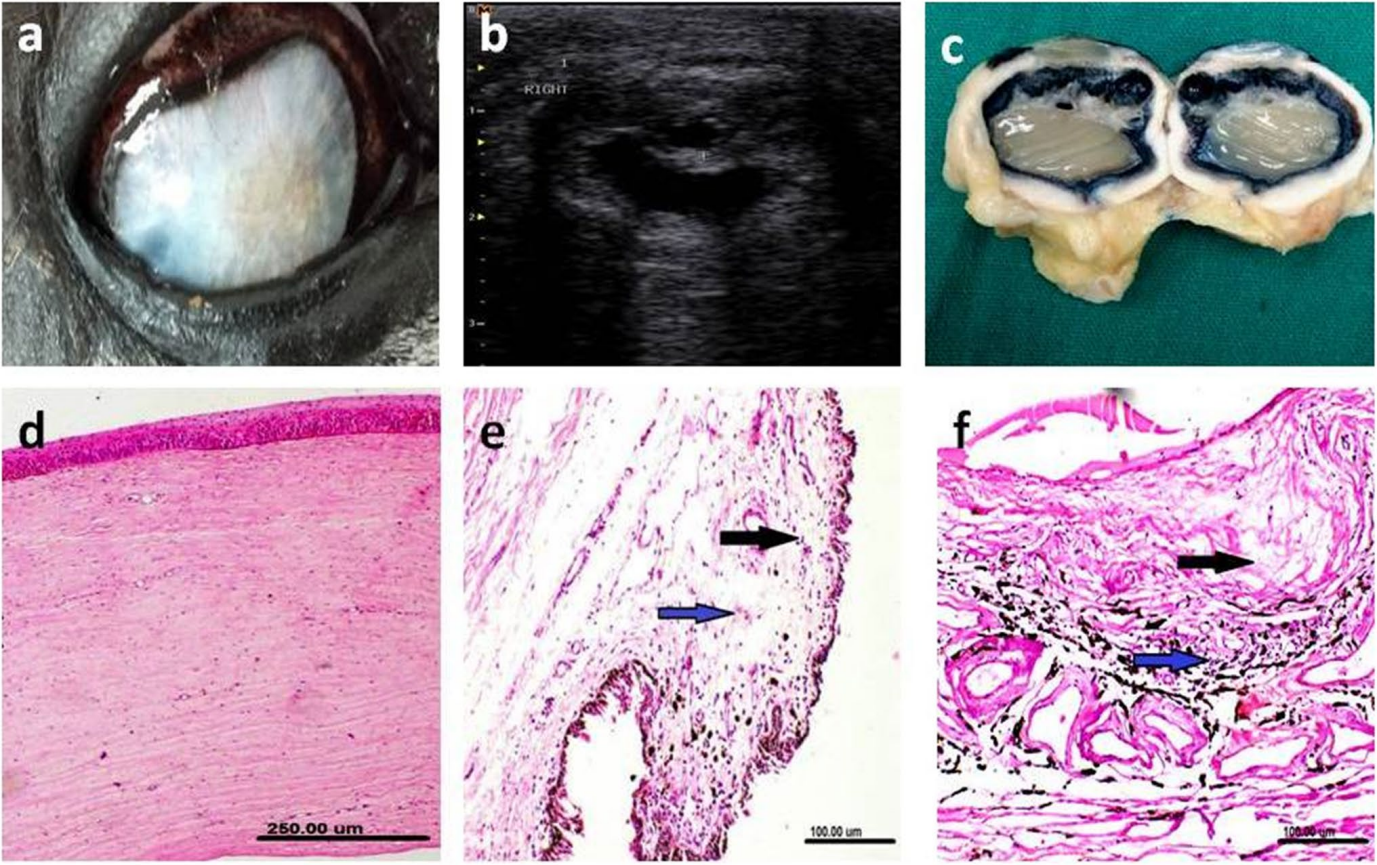
Clinical (**a**), ultrasonographic (**b**), gross pathologic (**c**) and histopathologic (**d**-**f**) changes associated with endophthalmitis that was progressed to phthesis bulbi. **a** The diseased eye showing whitish materials in the anterior chamber. **b** Ultrasonogram of the same eye showing echogenic material occupying most of the globe and thickening of the posterior wall of the eye. **c** Cross section of the same eye showing marked decrease in the globe size with presence of whitish material occupying the globe. **d** Histopathology of the cornea of the same eye showing marked thickening of corneal stroma. **e** The iris showing edema (blue arrow) associated with inflammatory cell infiltration (black arrow). **f** The choroid showing marked edema (black arrow) associated with inflammatory cell infiltration (blue arrow)

**Fig. 10:**
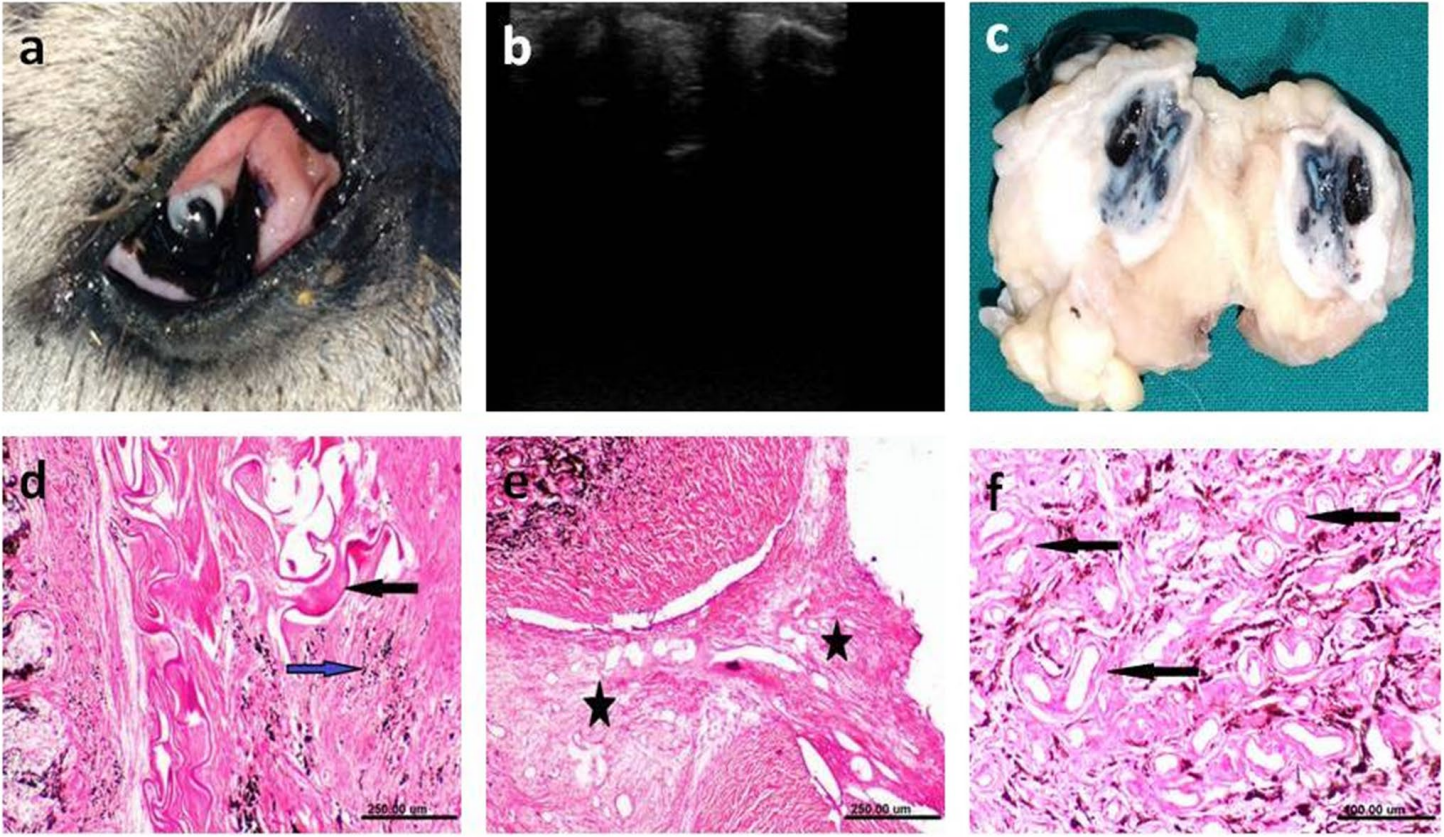
Clinical (**a**), ultrasonographic (**b**), gross pathologic (**c**) and histopathologic (**d**-**f**) changes associated with progressive phthisis bulbi. **a** The donkey showing enophthalmos, and non-functional eye with a prominent nictitating membrane. **b** Ultrasonogram of the same eye showing loss of detailed internal ocular structures with replacement of these structures by echogenic material. **c** Cross section of the same eye showing absence of the eye’s detailed structures. **d**-**f** Histopathological sections of the same eye showing: **d** Congestion of blood vessels [black arrow] and marked edema (blue arrow), **e** masses of myxomatous collagenous tissue [black asterisks], **f** perivascular fibrosis and hyalinosis [black arrow]

Marked pain, conjunctival and episcleral injection and loss of vision were seen within the eye. The cornea exhibited edema and neovascularization (Fig. 9a). A prominent nictitating membrane that was stretched across the globe with marked hypotony was seen in one eye (Fig. 10a).

#### Ultrasonographic examination

Ultrasonography revealed profound thickening of uveal tissue, marked and diffuse echogenicity in the anterior and posterior segments. The anterior chamber’s aqueous humor was replaced with echogenic substance and the vitreous chamber decreased in size. The globe axial length was markedly decreased (Fig. 9b) with presence of hyperechoic material obscuring and replacing the globe’s internal structures (Fig. 9c).

The detailed internal ocular structures were replaced by echogenic tissues in one eye (Fig. 10b) that was confirmed during postmortem examination by loss of the architectural details of the eye with marked reduction in globe size (Fig. 10c).

#### Histopathologic examination

Marked thickening and edema of the corneal stroma and inflammatory cell infiltration (mononuclear cell infiltration mainly lymphocytes and macrophages) in the iris were observed. Edema, inflammatory cell infiltration and perivascular hyalinosis were also observed in the choroid (Fig. 9d-f). Dark eosinophilic fragmented lens fibers and focal aggregation of macrophages laden melanin pigment were seen and associated with masses of myxomatous collagenous tissue, perivascular fibrosis and hyalinosis in the choroid (Fig. 10d-f).

## Discussion

The present study highlighted the role of ultrasonography as a valuable diagnostic tool offering insight into detailed intraocular structures. To the authors’ knowledge, this is the first study to include ultrasonographic findings of ocular diseases in donkeys with confirmed gross pathologic and histopathologic diagnosis. Studies presenting histological findings of ocular diseases are limited unless enucleation or exenteration are treatment options. The availability of enucleated eyes of donkeys with ocular diseases was very helpful and ethically approved to document the ongoing gross pathologic and histopathologic changes associated with ultrasonographic findings.

Most donkeys included in the present study were middle-aged donkeys; it could be assumed that theses donkeys were exposed to infectious diseases that has been missed or neglected by their owners, progressed to recurrent uveitis syndrome, ended up with complete loss of vision and termination of the working life. Recommendations should be directed towards increasing owner awareness to perform periodic screening of donkeys for early diagnosis and treatment of ocular diseases.

In the present study, trans-palpebral ocular scanning was used which was well tolerated by donkeys and did not necessitate the use of sedation or anesthesia with possible alteration on ocular hemodynamics (Scotty et al. 2004; Vrbovska et al. 2017).

Ultrasonographic characteristics of the normal eye of donkeys have been previously reported (Laus et al. 2013; Salavati et al. 2017; Wafy et al. 2021). However, no previous reports have been found to determine ultrasonographic features of ocular diseases in donkeys.

Studies comparing the ultrasonographic and histologic examinations of ocular diseases usually included small number of animals and the result of such comparison was not the main study outcome (Bentley et al. 2003). Reviewing veterinary literature, only one study has been published comparing ultrasonographic and histopathologic findings in a large number of animals (113 animals of different species including dogs, cats, and horses) (Gallhoefer et al. 2013),

In the present study, cataract was found to be the most commonly reported ocular pathology in donkeys (57 eyes; 76%) detected through clinical, ultrasonographic and histopathologic examinations. Similarly, cataract has been reported to be the most commonly recorded ocular pathology (57.1%) reported in a retrospective study retrieved from ultrasound scan of 112 horse’s eye (Scotty et al. 2004). On the other hand, a clinical study documenting the ophthalmic findings in a population of 205 donkeys in the UK revealed much lower rates where 41/205 (20%) of donkeys were diagnosed with cataract (Bradley et al. 2018) which could be attributed to the lower rates of infectious diseases inside the UK leading to uveitis and its sequelae.

A retrospective histologic study spanning 140 equine eyes revealed that non-traumatic keratitis represented the most commonly reported ocular pathology (25.7%) (Flores et al. 2020). While clinical and ultrasonographic survey study spanning 145 horses with visual impairment revealed that corneal alteration was the most commonly reported ocular pathology diagnosed in 83/145 (57.2%) eyes through clinical examination and 21/145 (14.5%) eyes during ultrasound examination (Gialletti et al. 2018). The disparity between results of different studies is partly due to different inclusion criteria and examination techniques of the examined eyes, and mainly due to the lack of high frequency ultrasound probe permitting visualization of the detailed structures of the cornea (Gialletti et al. 2018). The highest conventional (10–12 MHz) transducers do not allow detailed corneal examination compared to microscopic examination even with using stand-off pads (Scotty et al. 2004; Gialletti et al. 2018). Alternatively, transducers ranging from 20 to 60 MHz (biomicroscopy) can be used to improve corneal visualization at 20–80 μm compared to 300–400 μm at conventional 10 MHz transducer (Bentley et al. 2003).

In horses, it has been reported that cataract-related lens opacities are seldom to cause visual impairment until complete or extensive cortical opacification occurs (Matthews 2000). This could be mainly attributed to the wide horizontal visual field of the horse’s eye, the spatial arrangement of retinal receptor field, the absence of fovea and to the relatively high ganglion cell- photoreceptor ratio (Farrall and Handscombe 1999).

Descriptive clinical classification of cataract has been previously described in horses including both acquired (secondary) cataract, and congenital (developmental) cataract (Matthews 2000). Although little is known about the mechanism of cataractogenesis in the equine species especially in donkeys, sharing the same phylogenic mammalian origin and interspecies similarity of organization of mammalian lens makes the physicochemical and biochemical changes of cataract similar in most mammals (Matthews 2000).

In the present study, anterior uveitis was associated with cataract in all cases. It has been reported that the ongoing inflammatory changes within the uveal tract disrupts the blood-ocular barrier with subsequent protein and leakage into the surrounding connective tissue surrounding uveal tract (Bradley et al. 2020) which was manifested clinically by the associated aqueous flare, vitreal opacity and hypopyon. This exudation in turns decreases aqueous humor production by the ciliary body. Inflammatory mediators result in spasm within the ciliary and iris sphincter which was manifested clinically by as miosis (Bradley et al. 2020).

Disruption of the blood aqueous barrier disrupts the tight junctions of intercellular spaces of the non-pigmentary ciliary epithelium results in deposition of hyaline-like amyloid proteineous material lining the ciliary body (Cooley et al. 1990; Østevik et al. 2014).

The long-standing inflammation, alterations in aqueous humor together with synechiae formation may result in cataract formation.

A direct correlation between lens echogenicity and the degree of cataract has been documented with ultrasonography (Diaz 2004). Four ultrasonographic criteria have been used to diagnose cataract including: visualization of the anterior and posterior crystalloid even with absence of alterations in lens echogenicity; change of lens shape which tend to be rounded in shape; change in lens volume which tend to increase in thickness; and change in lens echogenicity which is commonly reported (Valentini et al. 2010).

Similar to previous reports, ultrasonographic diagnosis of cataract is usually associated with multiple abnormalities within the same eye (Scotty et al. 2004). In the present study, it was difficult to precisely determine the course of cataract with the presence of multiple involvements within the same eye as the donkeys were examined without complete case history.

Cataract was associated with vitreous opacities represented as hyperechoic linear or curvilinear echogenicities of various sizes and shapes which could be hemorrhage, white blood cells, asteroid hyalosis, or fibrin (Hallowell and Bowen 2007; Valentini et al. 2010). It is very crucial to perform ultrasonographic examination of curvilinear echogenicities at different scan planes to avoid misinterpretation of such echogenicities as retinal detachment (Valentini et al. 2010). In complete retinal detachment, the retina appears as hyperechoic line floating within the vitreous and still attached to the optic nerve (sea gull appearance).

In the present study, posterior lens luxation was identified ultrasonographically in two eyes where hyperechoic cataractous lens was displaced from its zonular attachment and seen freely in the vitreous. Lens luxation seems to be uncommon finding in horses; a previous study recorded 5 eyes with lens luxation in an 18-year study period (Scotty et al. 2004). Four of these lenses were cataractous (three uveitis-induced cataract and one traumatic cataract) and the fifth lens was non-cataractous, glaucomatous eye (Scotty et al. 2004). A posteriorly luxated, cataractous lens was identified in one out of 204 racing Thoroughbred horses during routine ophthalmic examination (Hurn and Turner 2006) and in one pony associated with glaucoma that was thought to be secondary to equine recurrent uveitis (McCluskie et al. 2009).

Limitations of the present study may include the relatively low number of eyes recorded with ocular diseases that limited the statistical analysis to determine the diagnostic accuracy of ultrasonography in detecting ocular pathology. The absence of complete historical data of examined donkeys that if present, would be helpful in understanding the onset, course and pathogenesis of the recorded ocular diseases.

## Conclusion

Ocular ultrasonography provided a valuable diagnostic tool offering insight into detailed intraocular structures. It was of great advantage when opacification of the dioptric structures hindered ophthalmoscopic examination. The study included ultrasonographic findings of ocular diseases in donkeys with confirmed gross pathologic and histopathologic diagnosis. Cataract represented the most common ocular pathology in donkeys with diverse effects on different ocular structures.

## Data Availability

The datasets generated during and/or analysed during the current study are available from the corresponding author on reasonable request.
